# Transgenerational loss and recovery of early learning ability in foraging predatory mites

**DOI:** 10.1007/s10493-017-0122-1

**Published:** 2017-04-13

**Authors:** Marliza B. Reichert, Inga C. Christiansen, Michael Seiter, Peter Schausberger

**Affiliations:** 1 Group of Arthropod Ecology and Behavior, Department of Crop Sciences, University of Natural Resources and Life Sciences Vienna, Vienna, Austria; 2 Laboratory of Acarology, Centro Universitário UNIVATES, Lajeado, Estado do Rio Grande do Sul Brazil; 3 Department of Behavioural Biology, University of Vienna, Vienna, Austria

**Keywords:** Experience, Pollen, Predator–prey interactions, Spider mites, Thrips, Transgenerational effects

## Abstract

The ability to learn is ubiquitous in animals but highly variable within and between species, populations and individuals. Diet-related circumstances, such as diet quantity and quality can influence both long-term constitutive (genetic; by selection) and short-term operational (non-genetic; by the immediate circumstances) learning performance. Here, we scrutinized the causes of loss of learning ability, following multi-generational feeding on pollen, in a line of the predatory mite *Amblyseius swirskii*, which was previously well able to learn prey during early life, enhancing foraging later in life. We investigated whether, and, if so, how quickly, a transgenerational diet switch to live prey restores the early learning ability of foraging predatory mites. The first experiment shows that the early learning ability was restored after switching the diet of the pollen-fed predator line to live spider mites for two generations before conducting the behavioral assay. The second experiment reveals that offspring regained their learning ability if the diet of their mothers was switched from pollen to spider mites for 3 or 10 days before offspring production. Both experiments in concert suggest transgenerational, pollen-induced operational loss of learning ability in the predatory mite *A*. *swirskii*. Maternally-transmitted nutrient deficiency and/or maternally-induced epigenetic changes are the most plausible explanations for the pollen diet-induced loss of learning ability. Our study represents a key example for maternal diet-induced variation in learning ability.

## Introduction

Learning is a ubiquitous phenomenon in animals, both vertebrates and invertebrates (Alloway 1972; Papaj and Lewis 1993; Smid and Vet 2006), and also observed in plant-inhabiting predatory mites of the family Phytoseiidae (Schausberger 2007; Rahmani et al. 2009; Schausberger et al. 2010; Walzer and Schausberger 2011; Strodl and Schausberger 2012). Learning is defined as adjusted behavior to a changing environment following experience (Lincoln et al. 1998; Alcock 2005) and may affect every major life activity, such as feeding, reproduction and social interactions. The ability to learn is shaped by natural selection but its phenotypic expression is strongly context dependent. For example, diet-related circumstances, like availability, quantity and nutritional composition of food, can strongly influence the expression of learning (for vertebrates: Wu et al. 2003; Akman et al. 2004; Yanai et al. 2004; for invertebrates: Xia et al. 1997; Kawecki 2010). Accordingly, any observed variability in learning expression may be due to constitutive (genetic) shifts in learning ability and/or be due to contextual factors (operational, non-genetic). Cases in point for constitutive and operational diet-related variability in learning expression come from the fruit fly *Drosophila melanogaster*. Regarding constitutive variability, Mery and Kawecki (2003, 2004) compared two lines of flies originating from the same base population, a high-learning and a low-learning line, in various contexts. Under limited but not abundant food availability, individuals from the high-learning line showed a poorer larval competitive ability (Mery and Kawecki 2003) and laid fewer eggs (Mery and Kawecki 2004) than individuals from the low-learning line. Regarding operational variability, Xia et al. (1997) observed a diet-induced shift in learning expression in *D*. *melanogaster*. Two lines of flies were reared, one on a standard medium, and the other on Peking medium, which is low in proteins and minerals and high in carbohydrates. Flies reared on the standard medium were able to operant visual learning and memory formation in a flight simulator, whereas flies reared on the Peking medium were not. After transferring young flies of the two lines to the other medium, and rearing the progeny on the new medium, loss of learning ability was observed in flies originating from the standard medium within three generations. Flies formerly reared on the Peking medium regained their learning ability and memory formation within five generations after transferring them to the standard medium. The work by Xia et al. (1997) clearly demonstrated a causal relationship between multi-generational malnutrition and learning ability of fruit flies.

Here, we scrutinized the causes of an observed loss of learning ability in a pollen–reared line of the generalist predatory mite *Amblyseius swirskii* (Athias-Henriot) (Seiter and Schausberger 2016). *Amblyseius swirskii* can feed on different prey species and types, like spider mites, whiteflies and thrips (McMurtry and Croft 1997; Nomikou et al. 2001; Messelink et al. 2008; Arthurs et al. 2009), but can also survive and reproduce on plant-derived substances like pollen (Park et al. 2011; Goleva and Zebitz 2013; Nguyen et al. 2013). These predators can be mass-reared, usually on factitious food or other than target prey (Fidgett and Stinson 2009), and are commercially available for use in biological control of phytophagous pests in greenhouse crops (McMurtry and Croft 1997; Messelink et al. 2008). Several predatory mite species, including the focal animal of our study, *A*. *swirskii*, are able to learn during their early life phase. The most sensitive phase for learning is shortly after hatching, throughout the larval phase and the early protonymphal stage (Schausberger 2007; Schausberger et al. 2010; Christiansen et al. 2016; Seiter and Schausberger 2016; Schausberger and Peneder 2017). Larvae of *A. swirskii* are facultative feeders, protonymphs are obligatory feeders (Wimmer et al. 2008). Christiansen et al. (2016) found that *A*. *swirskii* is able to learn the cues of a given prey early in life in foraging contexts, like other predatory mites, e.g. *Phytoseiulus persimilis* and *Neoseiulus californicus*, do (Rahmani et al. 2009; Schausberger et al. 2010; Schausberger and Peneder 2017). Learning is prey-specific and thus represents imprinting or associative learning but not sensitization, which is unspecific (Rahmani et al. 2009; Schausberger et al. 2010; Christiansen et al. 2016). Learning prey has benefits for the predators later in life, such as shortened attack latencies and a higher egg laying rate on familiar prey, but also incurs physiological costs, which are evident in longer developmental times (Christiansen et al. 2016). Recently, we observed that one line of *A*. *swirskii*, which was previously well able to learn (Christiansen et al. 2016), exhibited a complete loss of their learning ability in foraging contexts after rearing them for approximately 50 generations on ‘Nutrimite’, a diet exclusively containing pollen of narrowleaf cattail *Typha angustifolia* L. (Seiter and Schausberger 2016). In contrast, a sister line originating from the same population, but reared on spider mites for the same period of time, maintained its learning ability (Seiter and Schausberger 2016). Individuals of the pollen–reared line were no longer able to learn thrips prey during the larval and early protonymphal stage to be later in life, as adults, able to more quickly attack thrips. Thrips-experienced predators needed as long for attacking thrips as naïve predators exclusively fed on pollen, not experiencing prey during early life (Seiter and Schausberger 2016). Since learning was examined in predators shortly after hatching, the loss of learning ability must have been mediated by transgenerational effects. One cause for the lost learning ability could be inadvertent laboratory selection (Mery and Kawecki 2003). A population consists of different genotypes, with some individuals genuinely being better learners than others. The non-learners must have some life history advantage, for example a higher egg laying rate or a faster development time, as for example shown for *D*. *melanogaster* (Mery and Kawecki 2003), to outcompete the learners in an environment without a learning advantage, such as when living amidst surplus easy-to-get pollen. Thus, when a population only receives pollen for several generations, the relative proportion of learners should decrease, due to poorer life history performance. Assuming laboratory selection, the lost learning ability of pollen–reared *A*. *swirskii* could be due to a trade-off between maintenance of neuronal tissue for learning and investment in life history traits. Laboratory selection represents a long-term phenomenon, i.e. constitutively lost learning ability, which could be reversed after a diet switch to live prey, as long as individuals with the ability to learn prey remain in the population. Alternative causes of the lost learning ability could be either nutritional deficiency (Xia et al. 1997) and/or epigenetic changes (Naninck et al. 2015), and thus represent a short-term phenomenon, i.e. lost operational learning ability. In the latter cases, the predators should regain their learning ability within a relatively short time after a transgenerational diet switch from pollen to live prey such as spider mites.

The goals of our research were to scrutinize (1) if *A*. *swirskii* regains its lost early learning ability following a multi-generational diet switch to live prey and, if so, (2) if and how quickly the predators regain their learning ability after a maternal diet switch to live prey. We designed two experiments, which in concert should allow discriminating between the two major pathways possibly resulting in loss of learning ability, long-term selection (constitutive) or short-term (operational) trans-generational nutritional deficiency and/or epigenetic changes.

## Materials and methods

### Experimental and prey animals, population origins and rearing

*Amblyseius swirskii* used in the experiments derived from a population originating from Koppert Biological Systems (Berkel en Rodenrijs, The Netherlands). Upon receipt in October 2013, the population was split into two lines: one line was reared on ‘Nutrimite’ (Biobest, Westerlo, Belgium), a diet exclusively consisting of cattail pollen *T*. *angustifolia* (subsequently called PO–PO line), and the other was reared on a diet of two-spotted spider mites *Tetranychus urticae* Koch (Tetranychidae) (subsequently called SM–SM line). Both lines were reared on separate artificial arenas until conducting the experiments in spring 2015. The artificial arenas consisted of acrylic tiles (15 × 15 × 0.2 cm), resting on water-saturated foam cubes in plastic boxes (20 × 20 × 6 cm). Water-saturated tissue paper wrapped around the edges of the tiles confined the arenas. The arenas were equipped with small cotton tufts under cover slips to provide shelter and oviposition sites for the predators (e.g. Christiansen et al. 2016; Seiter and Schausberger 2016). The PO–PO line was fed with ‘Nutrimite’ by dusting pollen onto the arena once per week. The SM–SM line was fed with spider mites in 2–3 days intervals, by brushing spider mites from infested bean leaves (*Phaseolus vulgaris* L.), using a mite brushing machine (BioQuip^®^, Rancho Dominguez, CA, USA), onto glass plates and from there onto the rearing arena. *Tetranychus urticae* was reared on whole bean plants *P*. *vulgaris* grown at room temperature 23 ± 2 °C and 16:8 h L:D photoperiod.

Prey used in the experiments were live and dead (killed by deep-freezing) first instars of Western flower thrips *Frankliniella occidentalis* Pergande (Thripidae). Dead thrips were offered to ease feeding on thrips by the juvenile predators—live thrips are difficult to grasp and kill—to enable reinforcement and thus associative learning. Thrips were reared on detached bean leaves of *P*. *vulgaris* (ca. 11 × 13 cm) placed upside down on a 1% agar solution in a closed petri dish (14 cm diameter). For ventilation, a circular opening (1 cm diameter) was cut into the lid and covered with gauze. To obtain first instars, adult thrips were randomly withdrawn from the stock population, reared on whole green bean pods inside glass jars, and transferred to a fresh bean leaf for 24 h for oviposition. After removing adult thrips, the petri-dish was stored in a climate chamber at 25 ± 1 °C, 65 ± 5% relative humidity (RH) and 16:8 h L:D photoperiod for 3.5 days. At that time most larvae had hatched, which were then either directly withdrawn and used in the experiment as live prey or placed into acrylic cages, using a fine brush, killed by deep-freezing at −18 °C for at least 2 h and used in the experiment as dead prey.

All predator rearing arenas, thrips rearing units and experimental cages were kept in climate chambers at 25 ± 1 °C, 65 ± 5% RH and 16:8 h L:D photoperiod.

## Experimental procedures

### Multi-generational diet effects (experiment 1)

The first experiment aimed at determining whether *A. swirskii* from the PO–PO line can regain their learning ability by a multi-generational diet switch. To this end, a third line of *A*. *swirskii*, which derived from the PO–PO line, but the diet of which was switched to spider mites, was established (subsequently called PO–SM line). Twenty gravid females from the PO–PO line were placed on a new leaf arena (as described above), marked with small water color dots on their dorsal shields and reared on spider mites until the gravid females of the F1 generation (having no color dots) appeared on the arena, that is, approximately 10 days after establishing the PO–SM line. Offspring of the F1 generation were used in the experiment.

To obtain even-aged eggs giving rise to the experimental individuals, ten gravid females were randomly taken from each line, PO–PO, SM–SM and PO–SM, and placed on new, separate small leaf arenas (50 × 50 mm) for oviposition and fed with both spider mites and pollen. After 24 h, eggs were collected, using a fine moistened brush, and placed singly into acrylic cages (15 mm diameter, 3 mm high) previously loaded with either pollen (to generate thrips–naïve predators) or two dead plus one live thrips larvae (to generate thrips-experienced predators). The cages were closed at the bottom with fine gauze and on the upper side with a microscope slide (Schausberger 2007). To warrant elevated humidity inside, the cages were stored on a grid above tap water in an open plastic box. Three lines and two types of diet early in life resulted in six treatments: PO–PO + pollen, PO–PO + thrips, SM–SM + pollen, SM–SM + thrips, PO–SM + pollen and PO–SM + thrips. After hatching, the predatory mites were allowed to contact and feed on thrips (thrips-experienced) or pollen (thrips–naïve) in the larval and early protonymphal stage. The small size of the cage warranted repeated encounters of thrips by the larval/protonymphal predators. The experience phase lasted until the predators reached the protonymphal stage and on average 2–3 days from placing the egg into the cage. For consolidation of their early prey experiences, the protonymphs were then transferred to a new cage, using a moistened red marten’s hair brush, and only fed with pollen until reaching adulthood. Depending on the developmental time, the consolidation phase lasted 3–4 days. After the mites had reached adulthood, their sex was determined and males were discarded. A male, randomly taken from the same line as the experimental female, was added to each cage containing an adult female. The mite couple remained in the cage with access to pollen and free water for 24 h. To provide free water, each cage was equipped with a strip of filter paper tightly attached to the gauze on the bottom of the cage on one end and reaching into tap water with the other end. After 24 h with the male, the behavioral assay took place. To this end, each gravid female was transferred to a new cage, which had been previously loaded with four live first-instar thrips. Cages were checked for the occurrence and number of killed thrips, feeding on thrips by the predators (i.e. when observing the predators actively sucking on prey), as well as eggs laid by the predators, in 20 min intervals for the first 3 h, again after another 4 h and again the next day, i.e. after 24 h. Each of the six treatments was replicated 18–21 times.

### Maternal diet effects (experiment 2)

The second experiment aimed at determining whether *A*. *swirskii* of the PO–PO line can regain their learning ability by a maternal diet switch. We compared two new lines of predators, the diet of which had been switched to spider mites for 3 or 10 days before the experiment, named PO–PO–3 SM and PO–PO–10 SM. Each line was established by placing 10–15 gravid females from the PO–PO line on a separate arena. Predators of both lines, PO–PO–3 SM and PO–PO–10 SM, were first fed on pollen for 7 days, and then transferred onto new arenas where they were only fed with spider mites for 3–4 days (PO–PO–3 SM) or 10–11 days (PO–PO–10 SM). Eggs produced by the predator females on the 3rd or 4th day (PO–PO–3 SM) or 10th or 11th day (PO–PO–10 SM) after transfer to the spider mite arena were used for the experiment. The eggs were singly placed into acrylic cages and the emerging juvenile mites subjected to the same pollen and thrips experience and consolidation phases, and mated females then subjected to the same bioassay, as described for the first experiment. Two lines and two types of early diet experience resulted in four treatments: PO–PO–3 SM + pollen, PO–PO–3 SM + thrips, PO–PO–10 SM + pollen and PO–PO–10 SM + thrips. Each treatment was replicated 19 or 20 times.

### Statistical analyses

Statistical analysis was carried out using SPSS 21 (IBM-SPSS, Armonk, NY, USA). In the first experiment, we used separate generalized linear models (GLM) to analyze the influence of predator line (PO–PO, SM–SM or PO–SM) and early thrips experience (yes/no) on (1) the attack latency of adult predator females (Gamma distribution, log link), (2) the number of individual feeding events on thrips (binomial distribution-counts of events, logit link), and (3) the number of eggs laid by each female (Gamma distribution using eggs + 1, log link), followed by pairwise LSD or Sidak tests, if needed, to separate predator lines. Attack latency was defined as the time elapsed until the predators successfully attacked and killed the first thrips larva (see Schausberger et al. 2010). Likewise, separate one-sided GLMs were used to assess whether early thrips experience shortened the attack latency of adult predator females within each predator line (PO–PO, SM–SM or PO–SM) (Gamma distribution, log link). We used generalized estimating equations (GEE) to analyze the influence of predator line (PO–PO, SM–SM or PO–SM) and early thrips experience (yes/no) on the cumulative number of consumed thrips over time (Poisson distribution, log link), and to analyze the influence of early thrips experience (yes/no) on the cumulative number of consumed thrips over time (Poisson distribution, log linear) within each predator line.

In the second experiment, separate GLMs were used to analyze the influence of early thrips experience (yes/no) and predator line (PO–PO–3 SM or PO–PO–10 SM) on (1) the attack latency of adult predator females (Gamma distribution, log link), (2) the number of individual feeding events on thrips (binomial distribution-counts of events, logit link), and (3) the number of eggs laid per female (Gamma distribution using eggs + 1, log link). Likewise, separate one-sided GLMs were used to assess whether early thrips experience shortened the attack latency of adult predator females within each predator line (PO–PO–3 SM or PO–PO–10 SM) (Gamma distribution, log link). GEE was used to compare cumulative consumption of thrips over time (Poisson, log link) as affected by thrips experience and line identity (PO–PO–3 SM and PO–PO–10 SM).

## Results

### Multi-generational diet effects (experiment 1)

**Fig. 1:**
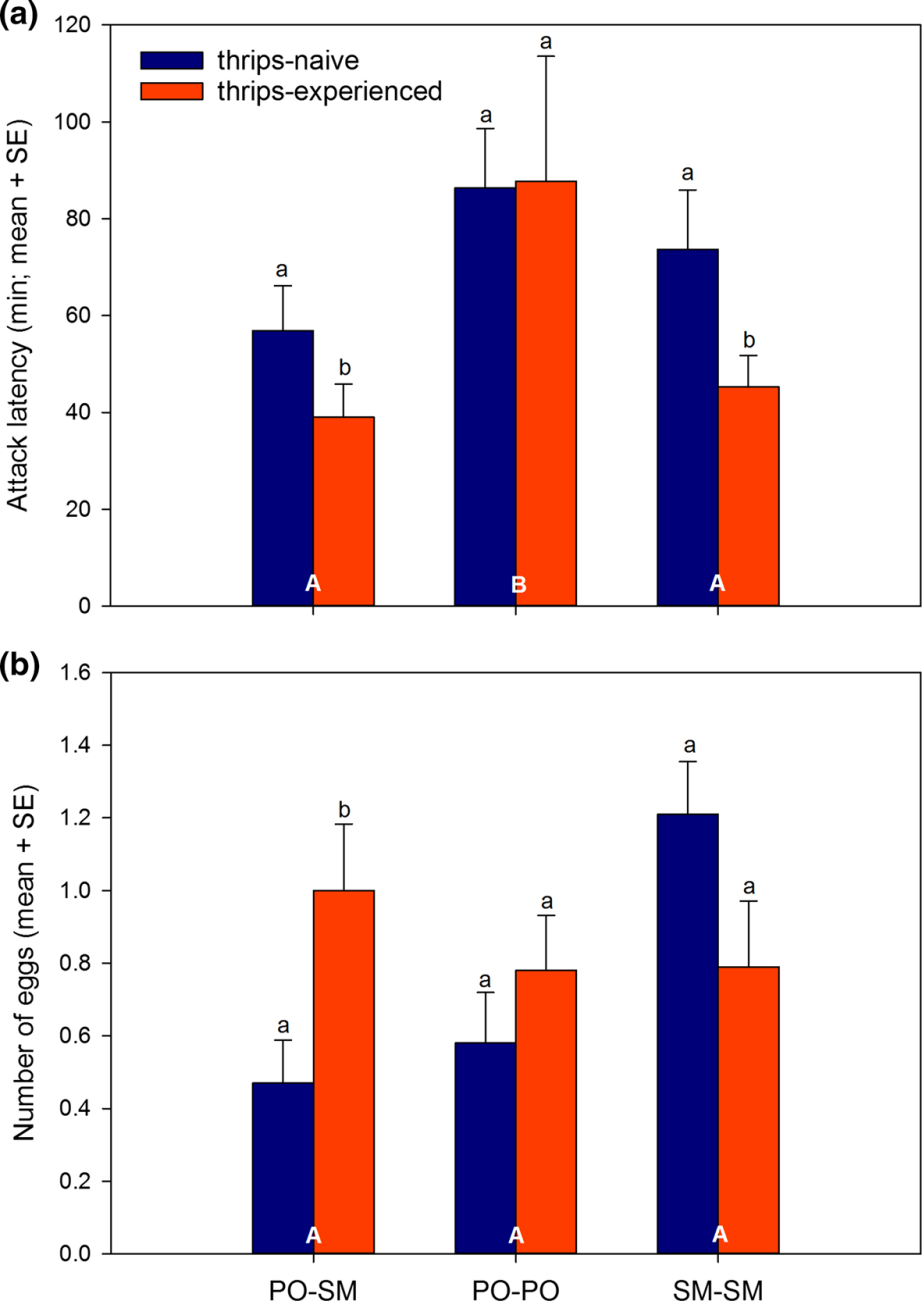
Attack latency and oviposition (experiment 1). **a** Time elapsed until attack on live first-instar thrips, **b** number of eggs laid by *Amblyseius swirskii* females (*N* = 18–21 per treatment of each line). The experimental animals were offspring from mothers from three distinct lines with different pre-experimental histories (PO–SM = pollen for 50 generations, then for one generation fed with spider mites *T. urticae*; PO–PO = pollen for 50 generations; SM–SM = spider mites for 50 generations). They experienced early in life, during the larval and early protonymphal stage, either thrips (experienced) or pollen (thrips–naïve) and were then fed only pollen until reaching adulthood. *Different upper case letters* inside pairs of *bars* indicate significant differences between predator lines (LSD following GLM; *P* < 0.05). *Different lower case letters* on top of *bars* indicate significant differences between thrips–naïve and thrips-experienced predators within predator lines (GLMs; *P* < 0.05)

**Fig. 2:**
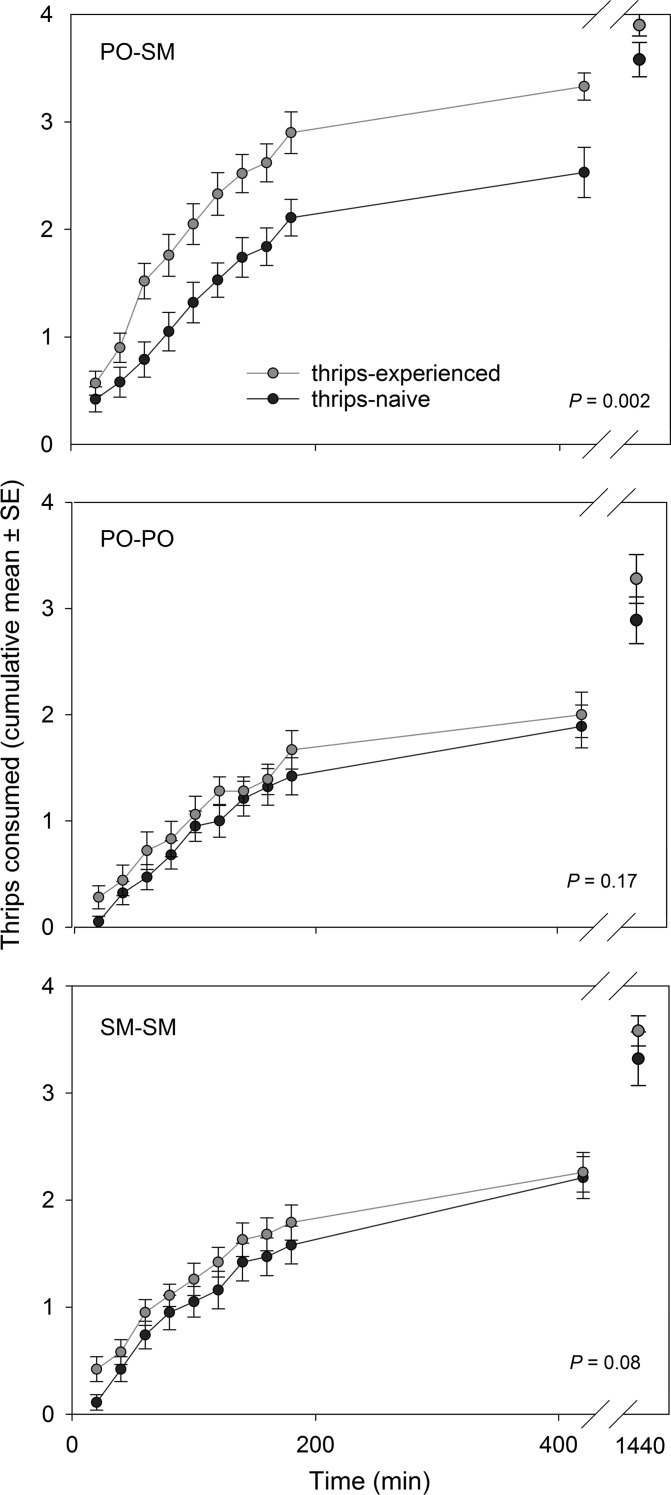
Consumption rate (experiment 1). Cumulative number of thrips consumed by adult *A. swirskii* females over time (*N* = 18–21 per treatment of each line). The experimental animals were offspring from mothers from three distinct lines with different pre-experimental histories (PO–SM = pollen for 50 generations, then for one generation fed with spider mites *T. urticae*; PO–PO = pollen for 50 generations; SM–SM = spider mites for 50 generations). They experienced early in life, during the larval and early protonymphal stage, either thrips (experienced) or pollen (thrips–naïve) and were then fed only pollen until reaching adulthood. *P* values inside graphs refer to GEEs comparing cumulative predation between thrips-experienced and thrips–naïve predators within each line over time

Thrips experience early in life (GLM; Wald χ_1_^2^ = 5.118, *P* = 0.02), in the larval and early protonymphal stage, as well as predator line (PO–PO, SM–SM or PO–SM; Wald χ_2_^2^ = 16.702, *P* < 0.001) affected the attack latency of adult *A*. *swirskii* females on first-instar thrips (Fig. 1a; Wald χ_2_^2^ = 2.944, *P* = 0.23 for the interaction). Predators from the PO–SM and SM–SM lines attacked thrips more quickly than predators from the PO–PO line (LSD: *P* < 0.05; Fig. 1a). Comparing the attack latency of *A*. *swirskii* on first-instar thrips within each of the three predator lines, revealed a significant influence of thrips experience in the SM–SM (GLM; Wald χ_1_^2^ = 6.181, one sided *P* = 0.005) and PO–SM (Wald χ_1_^2^ = 3.210, one-sided *P* = 0.04) lines but not in the PO–PO line (Wald χ_1_^2^ = 0.005, one-sided *P* = 0.47). Thrips-experienced predators from the SM–SM and PO–SM lines were faster in attacking thrips than thrips–naïve predators, which was not the case for predators from the PO–PO line. The mean number of individual feeding events on thrips by *A*. *swirskii* across time ranged from 0.50 ± 0.15 (SE) to 1.00 ± 0.22 and was neither affected by thrips experience early in life (Wald χ_2_^2^ = 0.018, *P* = 0.89), nor by predator line (Wald χ_2_^2^ = 1.194, *P* = 0.55) or their interaction (Wald χ_2_^2^ = 0.332, *P* = 0.85). Cumulative consumption of thrips by *A*. *swirskii* over time was affected by thrips experience early in life (GEE; Wald χ_1_^2^ = 12.270, *P* < 0.001) and predator line (Wald χ_2_^2^ = 33.328, *P* < 0.001) but not the interaction (Wald χ_2_^2^ = 1.087, *P* = 0.58) (Fig. 2). In all three lines, predators experiencing thrips early in life consumed more thrips as adult predators than thrips–naïve predators. Comparing cumulative consumption of thrips by *A*. *swirskii* within each of the three lines, revealed a (marginally) significantly higher thrips consumption by thrips-experienced than thrips–naïve predators in the PO–SM (GEE; Wald χ_1_^2^ = 9.969, *P* = 0.002) and SM–SM (Wald χ_1_^2^ = 3.137, *P* = 0.08) lines but not PO–PO (Wald χ_1_^2^ = 1.924, *P* = 0.17) line (Fig. 2). Neither thrips experience early in life (GLM; Wald χ_1_^2^ = 1.013, *P* = 0.31) nor predator line (Wald χ_2_^2^ = 4.589, *P* = 0.10) had a main effect on the number of eggs laid by *A*. *swirskii*, but the interaction was significant (Wald χ_2_^2^ = 9.315, *P* = 0.009) (Fig. 1b). Predators of all three lines laid on average between 0.5 and 1.2 eggs. In the PO–SM line, thrips–naïve predators laid fewer eggs than thrips-experienced predators (GLM; Wald χ_1_^2^ = 6.07, *P* = 0.01), whereas in the SM–SM (Wald χ_1_^2^ = 3.093, *P* = 0.08) and PO–PO lines (Wald χ_1_^2^ = 0.957, *P* = 0.33), thrips–naïve and thrips-experienced predators laid similar numbers of eggs (Fig. 1b).

### Maternal diet effects (experiment 2)

**Fig. 3:**
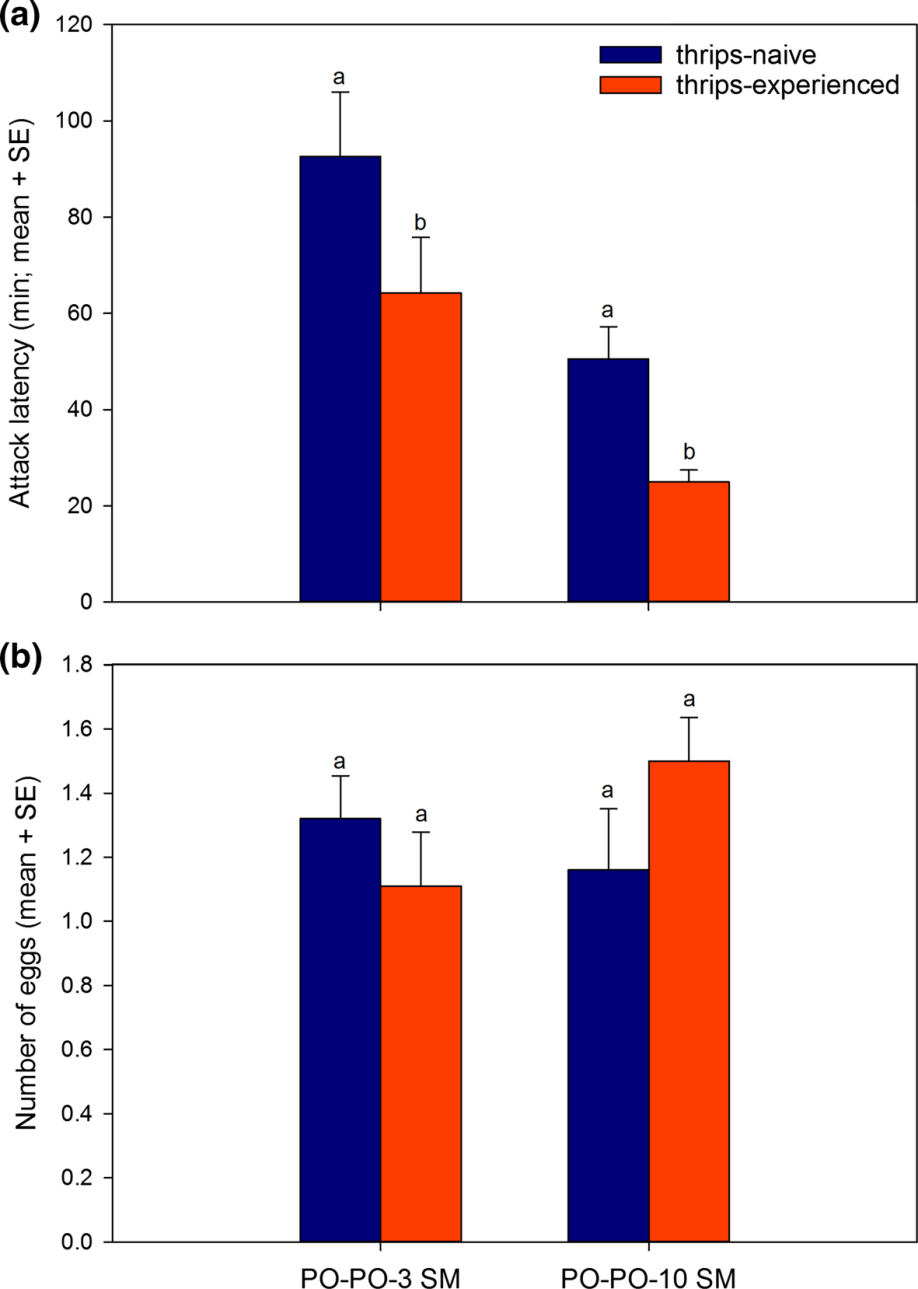
Attack latency and oviposition (experiment 2). **a** Time elapsed until attack on live first-instar thrips, **b** number of eggs laid by *A. swirskii* females (*N* = 19 or 20 per treatment of each line). The experimental animals were offspring from mothers that were formerly fed with pollen and then either for 3 (PO–PO–3 SM) or 10 (PO–PO–10 SM) days with spider mites *T. urticae*. They experienced early in life, during the larval and early protonymphal stage, either thrips (experienced) or pollen (thrips–naïve) and were then fed only pollen until reaching adulthood. *Different upper case letters* inside pairs of *bars* indicate significant differences between predator lines (GLM; *P* < 0.05). *Different letters* on top of *bars* indicate significant differences between thrips–naïve and thrips-experienced predators within predator lines (GLMs; *P* < 0.05)

**Fig. 4:**
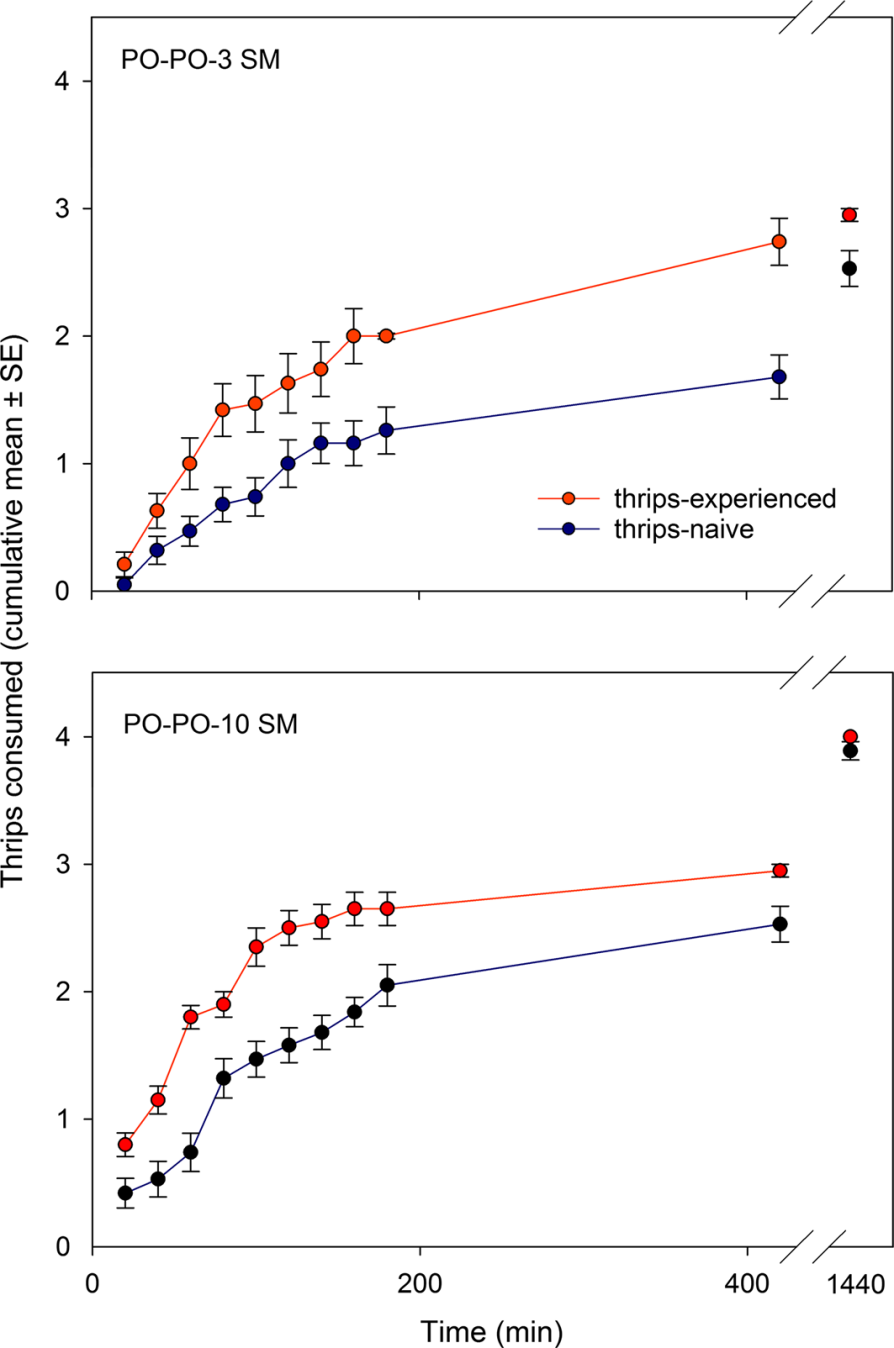
Consumption rate (experiment 2). Cumulative number of thrips consumed by adult *A. swirskii* females over time (*N* = 19 or 20 per treatment of each line). The experimental animals were offspring from mothers that were formerly fed with pollen and then either for 3 (PO–PO–3 SM) or 10 (PO–PO–10 SM) days with spider mites *T. urticae*. They experienced early in life, during the larval and early protonymphal stage, either thrips (experienced) or pollen (thrips–naïve) and were then fed only pollen until reaching adulthood. GEE revealed significant main effects of predator line (*P* < 0.001) and thrips experience (*P* < 0.001) but no interaction effect (*P* = 0.83)

Thrips experience early in life (GLM; Wald χ_1_^2^ = 16.715, *P* < 0.001), in the larval and early protonymphal stage, as well as predator line (Wald χ_1_^2^ = 35.044, *P* < 0.001) affected the attack latency of adult *A*. *swirskii* females on first-instar thrips (Fig. 3a) (interaction: Wald χ_1_^2^ = 1.659, *P* = 0.12). Thrips–naïve predators from both lines attacked thrips later than did thrips-experienced predators. Predators from the PO–PO–3 SM line attacked thrips later than did predators from the PO–PO–10 SM line, regardless if they had experienced thrips in early life or not. Within each of the two lines, thrips experience in early life significantly shortened the attack latency of *A*. *swirskii* on first-instar thrips (GLM; PO–PO–3 SM: Wald χ_1_^2^ = 3.082, one-sided *P* = 0.04; PO–PO–10 SM: Wald χ_1_^2^ = 19.668, one sided *P* < 0.001). The number of individual feeding events on thrips by *A*. *swirskii* across time was affected by predator line (Wald χ_2_^2^ = 3.734, *P* = 0.05), but not by thrips experience early in life (Wald χ_2_^2^ = 0.075, *P* = 0.79), and not by the interaction (Wald χ_2_^2^ = 1.219, *P* = 0.27). No matter if the predators experienced thrips early in life or not, predators from the PO–PO–10 SM line fed significantly more often on thrips than predators from the PO–PO–3 SM line (mean ± SE; 0.95 ± 0.12 vs. 0.58 ± 0.10 events per female). Cumulative consumption of thrips by *A*. *swirskii* was affected by thrips experience in early life (GEE; Wald χ_1_^2^ = 27.615, *P* < 0.001) and predator line (Wald χ_1_^2^ = 23.202, *P* < 0.001) but not by the interaction (Wald χ_1_^2^ = 0.048, *P* = 0.83) (Fig. 4). In both lines, thrips-experienced predators consumed more thrips as adult predators than did thrips–naïve predators. Predators from the PO–PO–10 SM line consumed more thrips than predators from the PO–PO–3 SM line. Neither thrips experience early in life (GLM; Wald χ_1_^2^ = 0.043, *P* = 0.84) nor predator line (Wald χ_1_^2^ = 0.189, *P* = 0.66) had an effect on the number of eggs laid by *A*. *swirskii* (Fig. 3b; Wald χ_1_^2^ = 1.128, *P* = 0.29 for the interaction).

## Discussion

Our study shows that offspring of *A*. *swirskii* females, which derived from a line that had been reared on pollen for >50 generations and had lost its foraging learning ability, regained their learning ability after multi-generational and maternal diet switches from pollen to spider mites. Full restoration of the learning ability of offspring occurred already after a 3 days-long maternal diet switch from pollen to spider mites. The time elapsed since maternal diet switch enhanced the overall predation performance of the offspring.

The first experiment shows restoration of the learning ability of the predatory mites after multi-generational diet switch from pollen to spider mites. Restored learning ability was evident in shorter attack latencies on first-instar thrips by adult predators after learning thrips in early life. The ability to learn prey in early life, resulting in faster recognition times and shorter attack latencies was shown before for *N*. *californicus* (Schausberger et al. 2010) and *A*. *swirskii* (Christiansen et al. 2016). Regarding cumulative thrips consumption, adult females from the PO–SM line consumed in total more thrips than predators from the PO–PO and SM–SM lines and, within lines, thrips-experience had the strongest effect on cumulative thrips consumption and egg production in the PO–SM line. Thus, predators from the PO–SM line not only regained their learning ability but profited the most from learning, as experienced predators produced significantly more eggs than naïve predators, which was not the case in the other two lines, PO–PO and SM–SM. However, in this context the physiological background of the lines regarding their efficacy in converting prey into offspring, deducible from eggs produced by naïve predators fed on live prey, needs to be looked at. It seems that the SM–SM line is physiologically superior to the other two lines in converting prey (animal food) into eggs, as evident from the high egg output by thrips–naïve SM–SM predators, which is likely a consequence of long-term rearing on prey and thus selection to favorably metabolize animal food. Experiment 2 suggests that the time elapsed since diet switch matters because the benefit of learning regarding egg output was only visible after a multi-generational, but not 3 or 10 days, maternal diet switch.

The second experiment more precisely determined the time period needed for restoration of the predators’ learning ability. Thrips-experienced predators produced by mothers, whose diet had been switched to live prey for 3 or 10 days before offspring production, attacked thrips more quickly, and consumed more thrips, than naïve predators, which is similar to the results of the first experiment. Revealing that *A*. *swirskii* can regain their learning ability by short-term maternal diet switches before offspring production, this experiment also suggests maternal effects on the overall vigor of the predators. The vigor of the predators apparently increased with the time elapsed since maternal diet switch, as indicated by the generally shorter attack latencies on the difficult-to-grasp prey thrips, and higher consumption rates of offspring from mothers switched to spider mite prey for 10 days than those switched for only 3 days. Other maternal effects described for predatory mites relate to foraging and predation risk contexts. For example, *P*. *persimilis* females, which are food-limited during internal egg formation, produce smaller-sized offspring (Walzer and Schausberger 2011); females stressed by intraguild predation risk during internal egg formation influence the anti-predator behavior of their offspring (Seiter and Schausberger 2015). Ultimately, it seems that mothers program their offspring differently, depending on the particularity of the diet regime experienced prior to egg production. Pollen–reared mothers switched to live prey for a brief period of time (PO–3 SM) adjusted their offspring less severely to live prey than mothers experiencing live prey for an extended period of time (PO–10 SM), which might be adaptive if the diet experienced by offspring is not persistently switched but remains variable. Proximately, the difference between lines switched for 3 and 10 days may also indicate that compensating for an alleged initial nutritional deficiency, due to pollen feeding, takes a couple of days, not for restoring learning ability but for increasing the vigor or adjusting the physiology of offspring to live prey. Alternatively, it may indicate that epigenetic changes (activating genes associated with predation and/or animal food) mediating adjustment to live prey take longer than changes needed to restore learning ability.

Early restoration, following the switch to live prey, of the learning ability of predators from the PO–SM lines in both experiments excludes laboratory selection as cause of loss of learning ability in the PO–PO line. Since laboratory selection can be excluded, restoration of the predators’ learning ability must respresent a nutritional (Xia et al. 1997) and/or epigenetic (Herb et al. 2012) maternal effect as underlying mechanism (Naninck et al. 2015). Assuming nutritional deficiency being responsible for loss of learning ability, pollen from *T*. *angustifolia*, as well as other plant-derived substances, might lack certain nutrients, which are necessary for learning, for example to strengthen existing, or establish new, neuronal connections. The nutritional value of pollen may differ from plant to plant and its digestibility for the animal as well (Roulston and Cane 2000; Lundgren 2009) but plant-derived food, as compared to animal food, often lacks, or contains in low quantities, certain nutrients that are important for proper functioning of the neural system such as B vitamins (especially B12, which is not present in plant but only animal tissue), or Omega 3 fatty acids such as docosahexaenoic acid (DHA), or essential amino acids such as tryptophane and phenylalanine, or minerals such as iron (Wakayama et al. 1984; Gomez-Pinilla 2008; Lundgren 2009; Bellen et al. 2010; Guesnet and Alessandri 2011; Kennedy 2016). Many plant-derived substances such as pollen are also suboptimal for life history traits, as compared to animal diets. For example, *A*. *swirskii* fed on the tomato russet mite *Aculops lycopersici* laid more eggs and developed more quickly than *A*. *swirskii* reared on cattail pollen (Park et al. 2011; Nguyen et al. 2015). Pollen of *T*. *angustifolia* contains 5–14% starch and up to 17% protein (Schmidt, Buchmann and Glaum 1989) and is only to a limited extent suitable for predatory mites. Nguyen et al. (2015) compared the life history parameters of the phytoseiid mites *N*. *californicus*, *Neoseiulus cucumeris*, *Amblyseius andersoni*, and *Amblydromalus limonicus* reared on *T*. *urticae*, *T*. *angustifolia* or on a liquid artificial diet consisting of honey, sucrose, tryptone, yeast extract and egg yolk, and showed that especially broad diet generalists, like *N*. *cucumeris*, *A*. *andersoni*, and *A*. *limonicus*, performed better on the artificial diet. Clearly, suboptimal nutritional diets, e.g. when *A*. *swirskii* feeds on pollen, may not only negatively affect life history traits but also cognition (for review: Gomez-Pinilla 2008; for rats: Yanai et al. 2004). In any case, pinpointing the nutrients lacking in *T*. *angustifolia* pollen and possibly being responsible for the loss of learning ability requires further scrutiny.

Assuming epigenetic mechanisms, genes, which need to be active for the process of learning prey, might be inactive in offspring from purely pollen-fed mothers. One of the first suggestions that DNA methylation affects gene expression and therefore regulates the on-and-off switching of genes during development was made by Holliday and Pugh (1975). For example, in the honeybee *Apis mellifera*, bees from different castes with different, distinct phenotypes share the same genotype (Herb et al. 2012). Workers and queens are irreversibly epigenetically programmed by diet during the larval stage regarding their caste affiliation, but with reestablished methylation levels for a majority of genes, foragers can be reverted to nurses, showing evidence for reversible, epigenetically controlled changes in behavior. Similarly, in our study, such epigenetic reversible changes are a possibility for restoration of the predators’ learning ability, possibly triggered by the smell, nutrients and/or behavior of prey. Nevertheless, these assumptions cannot be underpinned with studies dealing with epigenetics in phytoseiid mites, simply because such studies do not yet exist (Mukherjee et al. 2015). Recently, the genome of another phytoseiid mite, the Western orchard predatory mite *Metaseiulus occidentalis*, was sequenced (Hoy et al. 2016). Compared to other arthropods, it has a highly dynamic and turbulent genomic history, with a large number of intron losses and gains, making epigenetic regulation of various gene activities, such as those associaticed with pseudo-arrhenotoky (parahaploidy), very probable (Hoy et al. 2016).

The results of our study are of importance for the use of predatory mites in biological control. Predatory mites are commonly reared on factitious food or other prey than the target pest (Fidgett and Stinson 2009). Commonly, sachets containing mixed life stages of predatory mites are applied in greenhouse crops and the released individuals are target prey–naïve and might have impaired learning abilities, with possible unfavorable effects on biological control. This situation might be remedied by taking care that the composition of the food used in mass rearing does not compromise the predators’ learning ability, allowing them to learn and quickly adjust to the target prey after release.
